# ‘I know it has worked for millions of years’: the role of the ‘natural’ in parental reasoning against child immunization in a qualitative study in Switzerland

**DOI:** 10.1186/s12889-015-1716-3

**Published:** 2015-04-12

**Authors:** Karin Gross, Karin Hartmann, Elisabeth Zemp, Sonja Merten

**Affiliations:** Swiss Tropical and Public Health Institute, Socinstrasse 57, 4051 Basel, Switzerland; University of Basel, Basel, Switzerland; Children’s Hospital Lucerne, Lucerne, Switzerland

**Keywords:** Immunization, Vaccination, Childhood diseases, Immune system, Nature, Switzerland, Qualitative methods

## Abstract

**Background:**

Despite efforts of international and national health authorities, immunization coverage and timeliness of vaccination against dangerous childhood diseases have been adversely affected by parental hesitation to vaccinate their children in high-income countries. Literature shows that social and political processes and shifts in conceptual structures, such as emerging views linked to health and ‘natural’ lifestyles, have shaped parents’ immunization decisions. This paper investigates how Swiss parents argued along the lines of a natural development of the child to explain their critical attitudes towards immunization against measles and other childhood diseases.

**Methods:**

A total of 32 semi-structured interviews were conducted with parents of children between 0 and 16 years of age who decided not to fully immunize their children. The interviews were analyzed using qualitative content analysis and an interpretative approach.

**Results:**

Parents built their arguments against immunization on a strong faith in the strength of the naturally acquired immune system. Childhood diseases were not perceived as a threat but as part of the natural way to reinforce the body and to acquire a “natural” and thus strong immunity. Parents understood immunization as an artificial intrusion into the natural development of the immune system and feared overloading the still immature immune system of their young children and infants through current vaccination schemes.

**Conclusions:**

In the context of emerging trends towards natural lifestyles and ideas of holistic health in Switzerland and Europe, where many well-informed parents express concerns towards vaccinating their children, public vaccination strategies require reconsideration. Public immunization schedules need to acknowledge parents’ wish for more flexibility and demand for an individualized patient-centered approach to immunization.

## Background

Despite efforts of international and national health authorities, immunization coverage and timeliness of vaccination against dangerous childhood diseases have been adversely affected by parental choice to decline childhood vaccination in high-income countries [1-3]. This led to large-scale outbreaks of vaccine preventable diseases in Europe and the US such as measles in the Netherlands, Italy, France, Germany, Switzerland and the UK over the last ten years [4]. In Switzerland, after several years with a relatively low annual incidence rate, there have been several waves of large measles epidemics since 2006 [4,5]. As a consequence of these outbreaks and increased population awareness, coverage rate for two MMR doses among 2-year old children has slightly increased since 2005 to 86% [6], which is still considerably below the WHO target of 95% coverage. Moreover, coverage rates vary strongly between language zones and cantons ranging from 50 to 93% [7].

Recent studies argue that in today’s industrial countries vaccine refusal rates can only partially be explained by parents’ information deficit, lack of access to facts and misinformation [8,9]. Instead recent social-scientific literature has emphasized the importance of identifying broader social, political and conceptual structures that may shape parents’ immunization decisions in order to better understand reasons behind parental resistance to immunize their child [10]. Several authors see the reason behind parental hesitation to vaccinate their children as a combination of an increasing erosion of trust in stakeholders involved with vaccination and a parallel trend of an increasing personalization/individualization of risk [9,11]. However, in the last decade several studies have also reported on vaccination refusal among social groups having “alternative” lifestyles and philosophical outlooks [12-14]. Streefland [15] shows that there is a trend particularly among well-educated parents in Europe and the USA to believe in keeping good health by maintaining bodily balances and gaining immunity in a natural way. In his analysis of recent anti-vaccination movements Blume [1] also points to the emerging questioning of immunization among parents who advocate holistic ideas about health, natural child birth and breast feeding. Several studies support his observation: In a study exploring how parents in Brighton were thinking about the MMR vaccine in the context of the autism-controversy raised in the UK in the 1990s, several mothers backed up their arguments against MMR vaccination with the idea of the value of acquired natural immunity through nutrition and appropriate nurturing and the particularity of individual immunity [10,13]. Arguing that “alternative” views of health and medicine seem to be finding increasing sympathy among the population at large Blume [1] calls for a better understanding of them. Until now, there has been little formal research on these trends despite their influence on health care use [16,17].

In this paper we are especially interested in better understanding the influence of parents’ emerging views linked to “natural and healthy” lifestyles on their decision regarding childhood vaccination. This is particularly pertinent in Switzerland, where immunization costs are covered by insurance companies, but where vaccination coverage depends on parents’ initiative to bring their children to private pediatricians for vaccination. Based on the parents’ narratives we examined in particular how Swiss parents used themes around what they perceived as ‘natural development’ of the body and the immune system to explain their critical attitudes towards immunization against measles and other childhood diseases.

## Methods

### Study design

In order to explore parents’ perceptions and critical attitudes towards immunization against measles and other childhood diseases, semi-structured interviews were conducted with parents of children who had either been immunized incompletely (behind the recommended immunization schedule), partially (parents who chose to have only specific immunization) or who had not been immunized at all. We use the term *vaccine hesitancy* as described by Yaqub et al. [9] as all participants express critical attitudes towards vaccination but some may nonetheless vaccinate their children, although not according to the recommended schedule. In the interviews information on parents’ socio-demographic status and family composition was obtained. As part of an exploratory study on factors associated with vaccination resistance, the interviews addressed parents’ concepts of and attitudes towards childhood immunization, their individual experiences with vaccinations and the diseases, their perceptions of the risk, severity, and meaning of childhood diseases, their perceptions of immunization information disseminated by the media, doctors and the national health authority as well as their use of immunization services for their children. Since parents’ talking about the natural development of the body and the immune system emerged strongly from the interviews, we focus particularly on how Swiss parents used these themes to explain their critical attitudes towards immunization against measles and other childhood diseases.

### Recruitment

Parents were recruited from two cantons with high immunization coverage (Aargau (AG), Fribourg (FR)) and two cantons with low immunization coverage (Lucerne (LU) and Vaud (VD)) in the German and French speaking part of Switzerland [4]. To obtain a sufficient number of parents for the study different recruitment strategies were used: first, pediatricians and homeopaths working in the respective cantons were asked to inform and recruit parents. Since recruitment through this channel was difficult, participating parents were asked to provide further contacts, and finally two anti-vaccination organizations were contacted and asked for collaboration. Through this mix of purposeful sampling and snowball approach high heterogeneity among the participants was achieved.

### Data collection and analysis

A total of 32 interviews were conducted with parents who had children in the age range of 0 (youngest child) and 16 years of age (eldest sibling). All interviews were carried out in 2011 by a trained interviewer with a medical background (KH) in German and French. Interviews were conducted at parents’ home or a place that was convenient to them. An interview guideline that was pretested outside the study setting helped to structure the interviews. The average duration of the interviews was 60 minutes.

All interviews were audio-recorded and transcribed verbatim in Swiss German or French. The transcripts were analyzed with Atlas.ti using latent content analysis [18]. Text segments were coded and grouped by two independent researchers (KH and KG). There were no predefined coding themes; coding was entirely based on the content of the interviews. A final code catalogue was developed that guided the subsequent data analysis. The congruency of assigned codes and findings was continually compared during the data analysis process.

### Ethics and consent

Before the interview, informed consent was obtained from all participants. Parents were informed about the study and their right to stop the interview at any time. They were assured confidentiality and anonymity. Pseudonyms were used to anonymize the transcripts of the interviews. The study has been approved by the Ethics Committee of Basel (no. 346/10). The manuscript complies with the RATS guidelines for qualitative research.

## Results

### Sample characteristics

Out of the 32 interviews 28 interviews were conducted with the mother, one with the father and three interviews with both parents. Parents had a mixed socio-economic status but the majority could be characterized as middle-class with many parents working in the field of medical care (N = 19) or education (N = 9). Details on parents’ age, education and income are summarized in Table 1. Most of the families had two children. None of the interviewed parents had fully immunized their lastborn child. Nine parents had not immunized their lastborn child at all. Another 23 parents had only partially immunized their children or not yet immunized their lastborn child.

**Table 1:** **Participant characteristics**

**Characteristics**	**Total**		
	**n**	**%**	**mean**
**Interviewee(s)**			
Mother	28	88	
Father	1	3	
Both parents	3	9	
**Mean age in years**			
Mother			37
Father			40
**Highest education of mother**			
Obligatory education completed	0	0	
Secondary education completed	22	69	
University degree	10	31	
**Highest education of father**			
Obligatory education completed	6	19	
Secondary education completed	14	44	
University degree	12	38	
**Monthly household income**			
<5,000 Swiss francs	5	16	
5-10,000 Swiss francs	24	75	
>10,000 Swiss francs	3	9	
**Number of children**			
1	9	28	
2	14	44	
3	7	22	
≥4	2	6	
**Child immunization status**			
Fully immunized	0	0	
Not yet vaccinated	7	22	
Partially immunized	16	50	
Not immunized	9	28	

### The strength of the naturally acquired immune system

In the interviews, many parents built their arguments against immunization on a strong faith in the strength of the naturally acquired immune system. A mother working as acupuncturist argued for example that a strong immune system is able to cope with childhood diseases and cure them without the support of vaccines.

*“Our immune system is strong enough, and one should be able to overcome an illness with our own means” (VD01).*

Several parents supported their argument by referring to human evolution. There was a strong belief in the body’s perfect functioning, which has developed over millions of years and was contrasted with the vaccines’ short existence. A mother and nurse-in-training said for example,

*“I know it has worked for millions of years […]. I see this [vaccination] as antagonistic, because it [the vaccine] only exists for a few decades and it shall be much better than what has proved itself for millions of years; this does not make sense to me” (AG08).*

Parents generally described childhood diseases like measles, rubella, mumps or whooping cough as mild illnesses that many of them had gone through themselves as a child. Other childhood diseases such as polio were considered rare in Switzerland. Childhood diseases were, thus, not perceived as a threat but as part of the natural way to strengthen the body and to acquire “natural” immunity. Many parents, such as a mother working as a nurse specializing in sophrology (relaxation techniques to alleviate stress and tensions) presented diseases as meaningful and associated the experience of illness and recovery with progress in the child’s development.

*“Well, I think that it [illness] is like a necessary passage in order to grow. Often you see that children after overcoming a childhood disease make major steps in their development, acquisitions, and this again allows a good development to overcome these illnesses” (VD03).*

Illness was also perceived as a signal sent by the body in the case of tiredness or weakness forcing the person to rest and slow down. Parents argued that diseases come at the right time and are a more natural way to acquire immunity than through immunization.

*“If one is sick, it [the body] tells us… there is a message behind. The body tells us that we need to stop, to have a rest” (VD08).*

The non-immunized child’s health was often put in comparison with the health status of immunized children. Good health was taken as a reaffirmation.

*“We just really have a good feeling and we see, everything is fine with the kids, they are healthy. Healthier than other children in kindergarten, in comparison. And this is actually really a big affirmation for us that the way we are going is the right one. The right one for us” (AG03).*

Many parents argued that the strength of the immune system can be built up by healthy food. Moreover, breastfeeding was often mentioned as a sufficient measure to protect infants particularly in the early phase.

*“If one is really concerned with health, with food, with hygiene, with the whole life style, one has a natural immunity strong enough against so many things” (FR01).*

*“I think with breast milk a baby has already sufficient antibodies. Why inject then something else at such an early stage?”* (FR05).

### Immunization as an intrusion into natural bodily orders

Immunization was generally perceived as artificial and as an unnecessary intrusion into the development of a natural immune system and the healthy status of the child. Many parents, such as this mother working as a theater instructor, were convinced that the natural way of acquiring immunity through diseases should not be disturbed nor forestalled by a vaccine.

*“I have the feeling that I don’t want to meddle with it [natural immunity] and mess it up. Things exist and one has to handle them when they are there. But to treat an illness before it is there, ehm… that is something weird. It is like having a bypass operation while the heart is still strong” (LU02).*

Arguments against vaccination arise particularly in terms of the lower quality of vaccine-acquired immunity, the uncertainty about the bodily reactions to it, and its artificial timing. Protection through immunization was often not believed to be complete. Moreover, bodily reactions to a disease were considered different or even stronger when induced artificially compared to naturally acquired diseases. Many parents refused immunizations arguing as this mother and nurse *“that one is injecting something into the body without really knowing what it triggers”* (LU03). Parents expressed fears of upsetting the natural bodily order of the child or even impairing the child’s body. Those having small children below one year of age were particularly worried of “overloading” the immune system of their child with combined vaccines.

*“I have the feeling, one can overload the immune system with it, well, simply by giving the vaccine, or … yes, weaken the body somehow, so that it does not learn anymore to work well itself” (LU01).*

A mother working as a commercial employee for example interpreted the sickness she observed after the immunization as a sign that her child’s immune system was not able to cope with the immunization or was even further impaired by it.

*“With my older child I have waited for a whole year, and then I immunized him with polio, but I stopped afterwards. He reacted very strongly, he was afterwards constantly sick, well, he was permanently sick. The little one,… I have not yet vaccinated her for anything, well, I will not vaccinate anything, yes, because it does not make sense to me” (AG06).*

Some mothers pictorially described the combat of the body with the chemical substances of the vaccine, using notions of war and contamination.

*“I think one weakens the person, I say… by inserting something into the body that is something synthetic… that is nothing natural, hence, our body does automatically fight that, it does need to get used to it… and then, if one does not accept it… if the body does not accept it… They are a bit like bombs… I say for the children they are like bombs that are sent into their blood, that’s it (laughs)” (VD04).*

*“Well, yes, I think it is nevertheless a type of internal contamination and it does push our immune system, doesn’t it?” (VD05).*

A frequently raised concern was the artificial timing of the fight of the immune system with the substances of the vaccine. Even parents who planned to vaccinate their child opted for an individualized vaccination schedule and decided to postpone the vaccines “in order to allow their child to build up its immune system itself” (AG07).

## Discussion

Parents’ perceptions of the importance of a “natural” development of the body and the immune system emerged as a significant theme in explaining parents’ critical attitudes towards the immunization of their child(ren) against measles, mumps and rubella as well as other childhood diseases in Switzerland. Rather than simply “resisting” vaccination, this group of middle-class parents, a high proportion of which worked in the health and education sectors, showed a surprisingly strong faith in and appraisal of the “natural” development and functioning of the human immune system in their arguments. Earlier studies on parents’ vaccine decision-making in the UK have highlighted the close interconnection of parents’ decisions on natural birth, breastfeeding and vaccination [10,13,19]. Although not referring to natural ways of delivering, the interviewed parents in Switzerland similarly depicted worldviews emphasizing a particular notion of “the natural”. They repeatedly emphasized the important role of breastfeeding and healthy natural food to build up their children’s immune system. The health system itself has played an important role for the last two decades in promoting breastfeeding as the best way to protect an infant from infections through public health messages [20]. This may have contributed to a local culture of vaccination [21], which goes along with the underestimation of the need to vaccinate the child for specific diseases. In addition, recent campaigns to promote the consumption of particular foods such as fruits may support perceptions that a “natural” choice is a healthier choice. In our interviews, parents perceived diseases as a natural way to build-up a robust individual immunity, health and development, similar to what Leach and Fairhead described for the UK [10]. Our results support Blume [1] as well who has stressed the importance of considering parents’ ideas about holistic health and the importance of a natural life style and environment for a child’s well-being in order to understand parents’ vaccination decision-making.

One question, which emerges from these findings, is to what extent the preference for the natural over the artificial reflects a more deeply rooted distrust in the dominant bio-medical health care system. In their recent review on reasons for vaccine hesitancy in Europe Yaqub et al. identified distrust as a key barrier to vaccination, more precisely ‘distrust of doctors’, ‘distrust of government sources’, and ‘distrust of pharmaceutical companies’ [9]. The authors stressed that vaccines per se were not mistrusted, but the institutions delivering the vaccines. In some cases the motivation of health system actors was perceived as being influenced by larger economic interests. Our study differed in this respect in that distrust in institutions did not seem to be an overruling barrier to vaccination, albeit it was observed as well. But many of the interviewed parents worked themselves in the Swiss health care system, so that it is unlikely that they are overly critical of the system. Nonetheless they decided not to follow the official recommendations for vaccination.

Unlike in other studies our findings thus reveal a certain ambiguity as regards the importance of distrust of the health care system for vaccine hesitancy. Helpful for the interpretation of our findings is the conceptual distinction between trust and legitimacy. Yaqub et al. defined trust as ‘ability to rely on somebody else’s claims about a situation’ in the absence of full information, whereas legitimacy refers to the grounds (or discourses) upon which truth claims are made [9]. Among our respondents vaccine hesitancy seems to originate strongly in doubts about the legitimacy of an artificial intrusion into the “natural” development of the immune system rather than merely reflecting a general distrust in the actors of the health care system. When parents question the legitimacy of prioritizing the artificial stimulation of a child’s immune system over naturally acquired immunity, their uncertainty about bodily reactions to vaccinations might be part of “an increasing unease with modernity” which is paralleled by general concerns about the safety of mobile phones or environmental pollution, for example [17]. Helman postulated a general shift in the interpretation of the health-nature relationship: “In the 1970s . . . […] nature had become a threat. People were exhorted to keep it at bay, to protect themselves from the elements. In the new model, nature has come full circle and is once again valued as a positive, health-giving force (at least among the middle classes) (cited in [16]). Parents’ juxtaposition of naturally acquired immunity and health and their perception of vaccination as an artificial intrusion into the bodily order of the child refers well to what he described as part of a Western modern dualism endowing positive qualities to anything ‘natural’ and holistic as opposed to anything scientific or technical.

Besides doubts about the legitimacy of interfering with a “natural” process, fears about vaccination side effects played a role as well. Similar to what has been described by other authors, parents in Switzerland feared that vaccines combining several antigens could potentially overwhelm or “contaminate” the child’s immune system, even more so in the case of the still immature immune system of an infant [2,3,19]. Emily Martin described this phenomena already 20 years ago as a paradigm shift from a general concern of “disinfection” to “detoxification” [22]. Parental concerns of “overloading” their infants’ immune system with vaccines in the first months of life further open up discussions about the “right” timing of vaccines. Current practice of “one size fits all” immunization plans and combined vaccines raises concerns about not meeting every individual child’s needs. The rationale for recommendations as regards the timing of a specific vaccination is based on immunological and epidemiological considerations, which are usually not known to parents. Their desire for a personalized, patient-centered approach to vaccination in terms of age of the child and timing of vaccination may be accommodated with more precise information in this regard. A clear rationale for the recommended timing may motivate parents to follow the immunization schemes; in cases where flexibility is possible this could be communicated.

It is further notable that despite national information campaigns, the majority of interviewed parents associated childhood diseases such as measles, rubella and mumps with mild illnesses that many of them had gone through themselves when they were children, which was reported by other studies as well [2,23]. This illustrates again the very personalized perspective of parents when judging the risks and benefits of immunization versus disease [24]. Moreover, it points to the fact that risk perception always depends on the available possibilities to counter specific threats, such as the availability of a high quality curative health system [25]. In the Swiss context, contracting an infectious disease seemed to constitute a smaller threat to these parents than the uncertainty of the child’s bodily reaction to the vaccine.

We limited the focus of this paper on parents talking about nature, body and health as this reflects important lifestyle concepts in Switzerland and other high-income countries, which have up to this point received little attention in public health campaigns promoting vaccination. Decision-making on immunization involves many more dimensions, including parents’ understanding of their responsibility, their trust in health professionals and authorities, parents’ own health history, and social relationships with other mothers and parents [13]. Moreover, the small sample of parents critical to fully immunize their children cannot be generalized to the Swiss population. Nonetheless, our results provide important insights into parental reasoning on immunization, which may help to better adapt public health and vaccination messages.

## Conclusions

In the context of the Swiss health system vaccination coverage depends on parents’ initiative to take their child to a private pediatrician. Our findings suggest that well-informed parents make health-related decisions for their children on the basis of their own assessment, informed by their own worldview rather than basing it on experts’ recommendations alone. Unless emerging trends and lifestyles, such as the trend towards a more “natural” way of life, are addressed in vaccination campaigns, they might miss the target population. A related aspect is parents’ wish for a more personalized timing of vaccinations. To satisfy the increasing parental demand for an individualized, patient-centered approach to vaccination, a more flexible immunization plan, where possible, might help to increase immunization coverage. Moreover, national vaccination campaigns need to be integrated in a meaningful way into other public health messages that promote ‘natural’ ways to develop the human immune system, such as exclusive breastfeeding, to avoid seemingly contradictory messages. However, most importantly, there is a need for more research on the issue.
